# Persisting mortality gap in systemic lupus erythematosus; a population-based study on juvenile- and adult-onset SLE in Norway 1999–2022

**DOI:** 10.1093/rheumatology/kead519

**Published:** 2023-09-28

**Authors:** Sigrid Reppe Moe, Hilde Haukeland, Cathrine Brunborg, Antonela Botea, Nenad Damjanic, Gro Årthun Wivestad, Heidi Øvreås, Thea Bøe, Anniken Orre, Torhild Garen, Vibke Lilleby, Sella A Provan, Øyvind Molberg, Karoline Lerang

**Affiliations:** Department of Rheumatology, Oslo University Hospital, Oslo, Norway; Institute of Clinical Medicine, University of Oslo, Oslo, Norway; Department of Rheumatology, Oslo University Hospital, Oslo, Norway; Institute of Clinical Medicine, University of Oslo, Oslo, Norway; Department of Rheumatology, Martina Hansens Hospital, Gjettum, Norway; Oslo Centre for Biostatistics and Epidemiology, Research Support Services, Oslo University Hospital, Oslo, Norway; Department of Rheumatology, Betanien Hospital, Skien, Norway; Department of Rheumatology, Martina Hansens Hospital, Gjettum, Norway; Division of Rheumatology, Department of Medicine, Hospital of Southern Norway Trust, Kristiansand, Norway; Department of Rheumatology, Lillehammer Hospital for Rheumatic Diseases, Lillehammer, Norway; Department of Internal Medicine, Vestfold Hospital Trust, Tonsberg, Norway; Department of Rheumatology, Vestre Viken Hospital Trust, Drammen, Norway; Department of Rheumatology, Oslo University Hospital, Oslo, Norway; Department of Rheumatology, Oslo University Hospital, Oslo, Norway; Center for treatment of Rheumatic and Musculoskeletal Diseases (REMEDY), Diakonhjemmet Hospital, Oslo, Norway; Section for Public Health, Innland Norway University of Applied Sciences, Hamar, Norway; Department of Rheumatology, Oslo University Hospital, Oslo, Norway; Institute of Clinical Medicine, University of Oslo, Oslo, Norway; Department of Rheumatology, Oslo University Hospital, Oslo, Norway

**Keywords:** SLE, epidemiology, long-term outcome, mortality, sex, juvenile, childhood, lupus nephritis

## Abstract

**Objective:**

To estimate mortality and survival rates of SLE in a contemporary, population-based setting and assess potential influences by time, sex, ethnicity, classification criteria and age at diagnosis.

**Methods:**

We assessed mortality and survival in the Nor-SLE cohort, which includes all chart review–confirmed SLE cases resident in Southeast Norway (population 2.9 million) 1999–2017. Study end was at death, emigration or 1 October 2022. We defined juvenile SLE by age <16 years at diagnosis. For standardized mortality rate (SMR) estimates, we applied 15 population controls per case, all matched for age, sex, residency and ethnicity. We analysed survival by Kaplan–Meier and risk factors by Cox regression.

**Results:**

The Nor-SLE cohort included 1558 SLE cases, of whom 749 were incident and met the 2019 EULAR and ACR (2019-EA) classification criteria. SMR was increased to 1.8 (95% CI 1.6–2.2) in incident adult-onset SLE but did not differ between females and males. Survival rates at 5, 10, 15 and 20 years were lower in incident adult-onset SLE than in matched controls. In multivariable analysis, LN associated with decreased survival, while sex did not. Separate, long-term mortality analyses in the total Nor-SLE cohort showed that SMR peaked at 7.2 (95% CI 3.3–14) in juvenile-onset SLE (*n* = 93) and fell gradually by increasing age at SLE diagnosis.

**Conclusion:**

This study shows persistence of a mortality gap between adult-onset SLE and controls at population level and provides indications of worryingly high mortality in juvenile-onset SLE.

Rheumatology key messages

- The excess mortality in adult-onset SLE persists.
- Indications of a wide mortality gap in juvenile SLE is a major concern.
- Sex appears not to influence the width of the mortality gap in SLE.

## Introduction

SLE is a complex and heterogeneous multi-organ autoimmune disease with female predominance and reduced life expectancy [1]. SLE runs a chronic disease course characterized by fluctuating disease activity, organ damage accrual and excess risk of comorbidities, such as cardiovascular disease and infections [2].

Detailed and unbiased knowledge of long-term outcomes in SLE is crucial to understand the individual and societal burden of the disease and to identify risk factors for adverse outcomes [3]. The latter is important for clinicians as it may aid in the identification of patients with poor prognosis and a need for intensified surveillance and therapeutic interventions [4]. Unfortunately, it has proven challenging to study SLE at population level. A major reason is that SLE-specific International Classification of Diseases (ICD) codes set by specialists are considered to have low accuracy, which means that they are difficult to apply as proxy for SLE diagnosis [5]. Accordingly, for population-level studies of SLE, individual-level confirmation of diagnosis by expert clinical assessment appears necessary to achieve accurate knowledge, and it is time-consuming and costly. Most likely due to the costs and efforts required, the population-based studies who have applied ‘SLE diagnosis by expert clinical assessment’ as case definition are, as a rule, limited in cohort size and/or study duration, resulting in low power [4]. Accordingly, it has proven difficult to obtain sufficiently powered population-based data on how patient characteristics affects outcome. This is especially so for less common characteristics. To allow for a larger sample size, many population-based studies opt to include non-incident cases (with longer disease duration) in the outcome analyses [3]. Even though this approach inevitably introduces bias, particularly regarding survival, it may provide valuable information on how patient characteristics influence outcome [6].

Key examples of uncommon and difficult-to-study patient characteristics in SLE are male sex and juvenile-onset disease, each of which comprise about 10% of the total SLE population [7, 8]. Male patients appear to be at risk for severe disease with damage accrual, but it is not clear from population levels studies whether their prognosis is worse than that of female SLE patients [9]. Similarly, while reports indicate high risk of adverse outcomes, the long-term mortality in juvenile-onset SLE is not well described [10]. In fact, the mortality datasets that do exist are small, from tertiary-based centres or with follow-up <10 years [11–16].

In Norway, every citizen has equal access to healthcare through national, state-financed systems. The mandatory and unique 11-digit personal identification number of every Norwegian inhabitant make linkage to national registries possible and minimizes the risk of loss to follow-up of cases during observation. Patients with SLE are managed long term by specialists in public hospitals, which presents a unique opportunity to gather a complete SLE cohort capturing the whole spectrum and natural course of the disease.

The main objective of this study was to investigate long-term outcomes of SLE at population-level and assess possible differences in outcome between age groups, males and females, classification criteria and secular trends. We benchmarked mortality and survival, the key outcome measures, against general population controls individually matched to each case regarding sex, age, ethnic ancestry and residency. We focused the study primarily on incident SLE (i.e. the cases diagnosed from 1999 and onwards), but to allow for assessment of select patient characteristics, we also included targeted analyses of standardized mortality rates (SMR) in the total SLE cohort (non-incident and incident cases).

## Methods

### The population-based Nor-SLE cohort

For this study, we applied the population-based Nor-SLE cohort, which has been described in detail elsewhere and includes all cases with a confirmed SLE diagnosis who were resident in the Southeast Norway area during 1 January 1999 to 31 December 2017 (H.H. Haukeland *et al.*, submitted). By 1 January 2018, this study area had 2.9 million inhabitants: 56% of the total population in Norway. The area includes Oslo, the capital and largest city in Norway.

In Supplementary Fig. S1 (available at *Rheumatology* online), we review inclusion of cases in the Nor-SLE cohort. Briefly, we first identified all cases registered with an SLE-specific ICD 10th Revision (ICD-10) discharge code (M32.1, M32.8, M32.9) in the period 1 January 1999 to 31 December 2017 in the study area patient administrative databases. We conducted individual-level review of out- and inpatient hospital medical charts to confirm SLE diagnosis in line with the diagnostic principle of Fries and Holman [17]. We used the 11-digit personal identification number to control duplicate registrations and to identify cases with registrations in more than one hospital database. We retrieved patient demographics from Statistics Norway, who define ethnicity by parents’ country of birth (with offspring being Norwegian if at least one parent was born in Norway). Ethnic groups were Europeans (including Russia), Asians (including Turkey), Africans and South-and Central Americans. Nor-SLE cohort cases were defined as incident if they had new SLE diagnosis between 1 January 1999 and 31 December 2017, and were resident in the study area at the time of diagnosis or settled in the area within 1 year of diagnosis.

### Data collection

From individual patient-chart review, the Nor-SLE study group collected and recorded retrospectively clinical data at time of diagnosis, after 2 years’ disease duration and at last follow-up. Information included cumulative occurrence of the items in the 1997 ACR classification criteria for SLE (ACR-97) in all cases, and the items in the 2019 EULAR/ACR classification criteria for SLE (EA-2019) in the incident population. Presence of LN was defined by ACR-97 or EA-2019 criteria. We defined use of anti-malaria medication as ever or never used where ever used were defined as treatment for 6 months or more.

### Study design

We defined the study as a prospective population-based observational cohort study. For this study, the total SLE cohort included all adult and juvenile Nor-SLE cohort cases (incident and non-incident) who fulfilled the ACR-97 criteria. Follow-up started from the year of capture (1999 or later) ended at first occurring event of censor (1 October 2022), death or migration out of Norway.

For analyses of the adult and juvenile incident cases, we applied both the ACR-97 and the 2019 EA-2019 criteria to enable internal and external comparisons, resulting in overlapping cohort subsets who fulfilled the ACR-97 criteria (the incident ACR-97 cases) and the EA-2019 criteria (the incident EA-2019 cases), respectively.

### Controls

Controls were randomly drawn from the National Population Registry, 15 per SLE case, matched according to year of birth, sex, ethnicity and residency (county, as well as urban or rural area), and vital status at start of follow-up. Ethnic groups were defined as outlined above, but here we subdivided Asia in two categories: (i) East Asia (South, Central Asia and the Middle East including Turkey) and (ii) West Asia (South and Southeast Asia including China). Cases and controls were included at the same time in the study.

### Assessment

The National Cause of Death Register (NCoDR) includes all deaths in Norway and is based on death certificates. In October 2022 we obtained time of death on all cases and controls from NCoDR, and, if applicable, year of emigration.

### Statistical analysis

Categorical data were analysed by chi-squared test. Continuous variables are presented as means (s.d.) if normally and medians (interquartile range) if non-normally distributed. We examined survival by Kaplan–Meier estimates and tested for differences with log-rank test. To estimate risk of death, we performed calculations of SMR where the expected number of deaths referred to the number of deaths for the matched controls. The 95% CI of SMR was calculated with Mid-P exact test.

We stratified cases by age groups and defined (i) juvenile SLE as cases <16 years and (ii) adult-onset SLE ≥16 years at SLE diagnosis, where adult-onset was subdivided in (iii) early-onset (16–49 years) and (iv) late-onset (≥50 years). To investigate possible changes in survival during the study period, we divided the incident populations by year of diagnosis: (i) 1999–2008 and (ii) 2009–17.

A total of 32 SLE cases (3%) and 677 controls (3%) migrated out of Norway during the study period and were lost to follow-up but contributed with data until migration.

We performed uni- and multivariable Cox regression in both the ACR-97 and EA-2019 incident populations to investigate factors associated with survival. Multivariable analyses were performed for each factor separately adjusting for sex, ethnic ancestry, LN and age. The proportional hazard assumptions were tested by Schoenfeld tests and found to be satisfied. Harrell’s c-index was used to evaluate the factors discriminatory capability when adjusting for covariates. For technical reasons, we lacked complete follow-up data on 29 incident cases diagnosed in 1999 and therefore excluded all diagnosed in 1999 from the multivariable analysis.

### Ethics

This study was approved by The Regional Committee for Medical and Health Research Ethics with exemption from informed consent for identification of patients, chart review and linkage to NCoDR (REK 2009/2017).

## Results

### Study populations

Individual chart review–confirmed SLE diagnosis in 1558 of the 3488 individuals identified with a SLE-specific ICD-10 code 1999–2017. Of these 1558 cases with a confirmed SLE diagnosis, 797 had new-onset disease in 1999–2017, of which 749 (94%) fulfilled the EA- 2019 criteria (incident EA-2019 cases) (Table 1 and Supplementary Fig. S1, available at *Rheumatology* online).

**Table 1.** Demographic, clinical features and outcome parameter in SLE by case cohort and matched controls

	Incident cohort		Total cohort	Matched controls
Classification criteria applied	EA-2019^a^	ACR-97^b^	ACR-97^b^	n/a
Number of cases/controls	749	673	1300	19 500
Baseline demographics				
Age at diagnosis, years µ (s.d.)	39 (16.6)	38 (16.1)	35 (15.7)	n/a
Age at study inclusion, years µ (s.d.)	39 (16.6)	38 (16.1)	40 (15.7)	40 (15.7)
Female, *n* (%)	632 (84)	573 (85)	1123 (86)	16 845 (86)
Of European ancestry, *n* (%)	623 (83)	558 (83)	1141 (88)	17 115 (88)
Juvenile-onset^c^, *n* (%)	39 (5)	37 (5)	93 (7)	n/a
Early-onset^d^, *n* (%)	520 (69)	479 (71)	964 (74)	n/a
Late-onset^e^, *n* (%)	190 (25)	157 (23)	243 (19)	n/a
Clinical features				
LN^f^, *n* (%)	240 (32)	222 (33)	472 (36)	n/a
ANA positive, *n* (%)	749 (100)	666 (99)	1278 (98)	n/a
EA criteria^a^, score at study end, µ (s.d.)	22.5 (8.1)	23.3 (8.2)	n/a	n/a
ACR criteria^b^, score at study end, µ (s.d.)	5.0 (1.4)	5.3 (1.2)	5.4 (1.2)	n/a
Outcome parameters				
Follow-up years^g^, µ (s.d.)	13 (5.8)	13 (5.7)	16 (6.7)	17 (6.5)
Deaths, *n* (%)	106 (14)	83 (12)	301 (23)	2109 (11)
Disease duration at death, years µ (s.d.)	11 (6.1)	11 (6.1)	21 (12.5)	n/a
Age at death, years median (IQR)	72 (60–81)	72 (59–80)	69 (57–77)	75 (66–84)

In incident SLE cases (new-onset disease 1999–2017), we applied both the 1997 ACR (ACR-97) and the 2019 EULAR/ACR (EA-2019) classification criteria for SLE. In SLE cases living in the study area 1999–2017 (total cohort), we applied the ACR-97 classification criteria for SLE. Each SLE case were individually matched to 15 population controls by sex, age, residential area and ethnic ancestry.

a The EULAR/ACR classification criteria for SLE.

b The 1997 ACR classification criteria for SLE.

c Diagnosis before age 16 years.

d Diagnosis between the age of 16 and 49 years.

e Diagnosis ≥50 years of age.

f LN by the 1997 ACR or the EULAR/ACR classification criteria for SLE.

g From 1999 or, after 1999, from year of relocation to study area or year of diagnosis. *n*: number; µ: mean; IQR: interquartile range; n/a: not applicable.

### Mortality and survival in the incident SLE cases

At group level, the estimated mortality and survival rates for the incident cases who fulfilled the 2019-EA (*n* = 749) and the 97-ACR criteria (*n* = 673) were close to identical. Hence, for simplicity, we focus on the results for the incident EA-2019 cohort and refer to Supplementary Material for complete data sets on the 97-ACR cohort (Supplementary Tables S2 and S3, available at *Rheumatology* online).

The incident EA-2019 cohort (and the individually matched control population), consisted of 84% female and 16% male cases (Table 1). The male SLE cases were older at diagnosis, had a higher frequency of LN (50% *vs* 29%, *P*-value <0.001) and were more likely to be of European ancestry (91% *vs* 82%, *P*-value <0.001) than female cases (Supplementary Table S1, available at *Rheumatology* online). After mean 13 years of follow-up, 106 of the 749 cases (14%) in the incident EA-2019 cohort had died. All the 106 deceased cases had adult-onset disease (Table 2).

**Table 2.** SMR in new-onset SLE when compared with the matched controls, by patients characteristics and case cohort

	Incident cohort	Matched controls		Incident cohort	Matched controls	
Criteria applied	EA-2019^a^	n/a		ACR-97^b^	n/a	
Cases/controls, *n*	749	11 235		673	10 095	
	Deaths, *n*	Deaths, *n*	SMR (95 % CI)	Deaths, *n*	Deaths, *n*	SMR (95 % CI)
Total	106	911	1.8 (1.5–2.2)	83	722	1.7 (1.4–2.2)
Female	73	547	2.0 (1.5–2.5)	58	486	1.8 (1.3–2.3)
Male	33	367	1.5 (1.0–2.2)	25	236	1.6 (1.0–2.5)
Juvenile-onset^c^	0	3	n/a	0	3	n/a
Adult-onset^d^	106	911	1.8 (1.6–2.2)	83	722	1.7 (1.4–2.2)
Early-onset^e^	29	146	3.0 (1.9–4.5)	24	140	3.0 (1.9–4.7)
Late-onset^f^	77	768	1.6 (1.2–2.0)	59	582	1.6 (1.2–2.0)
Ancestry						
European	100	879	1.8 (1.4–2.2)	78	696	1.7 (1.3–2.5)
Non-European	6	35	2.6 (0.9–6.3)	5	26	2.8 (0.8–7.4)
LN^g^						
Absent	38	662	1.6 (1.2–2.0)	54	540	1.5 (1.1–2.0)
Present	68	252	2.4 (1.7–3.4)	29	182	2.5 (1.6–3.7)

In incident SLE (new-onset disease 1999–2017) we applied both the 2019 EULAR/ACR (EA-2019) and the 1997 ACR (ACR-97) classification criteria for SLE. Each SLE case were individually matched to 15 population controls by sex, age, residential area and ethnic ancestry.

a The EULAR/ACR Classification Criteria for SLE.

b The 1997 ACR classification criteria for SLE.

c Diagnosis before age 16 years.

d Diagnosis ≥16 years.

e Diagnosis between the age of 16 and 49 years.

f Diagnosis ≥50 years of age.

g LN by the 1997 ACR or the 2019 EULAR/ACR classification criteria for SLE. *n*: number; SMR: standardized mortality rate; n/a: not applicable.

**Figure 1.**
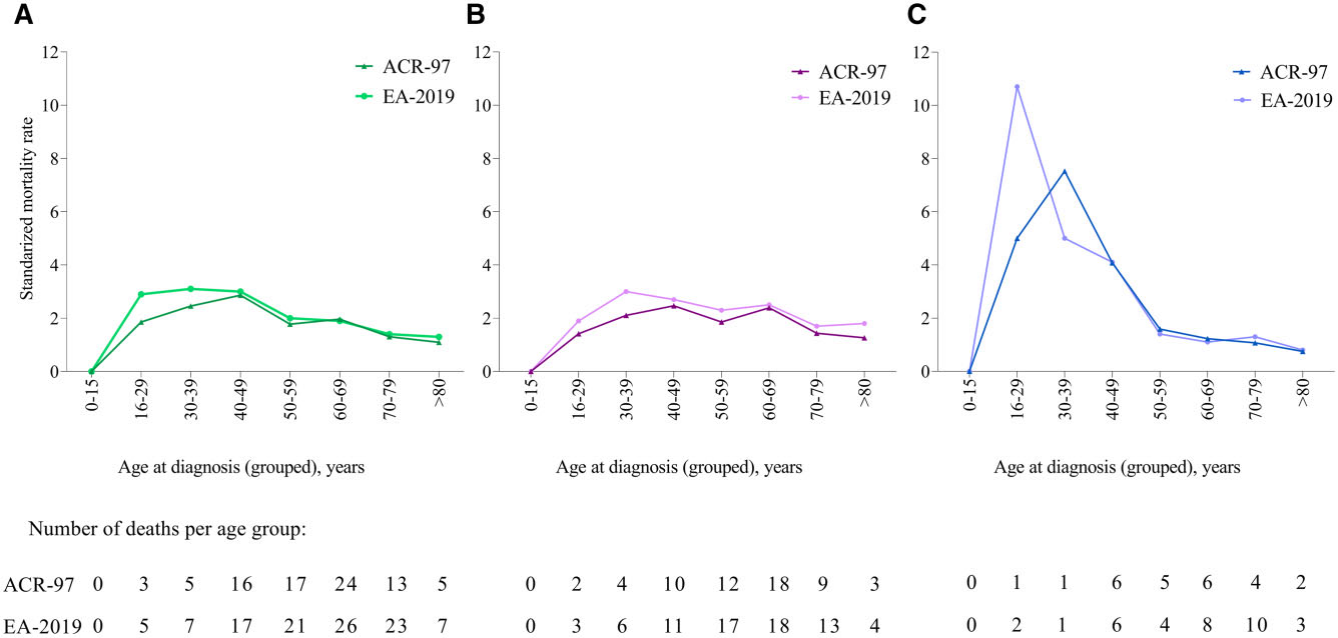
Age-specific SMR in new-onset SLE cases 1999–2017. Age-specific SMR in (**A**) all, (**B**) females and (**C**) males with disease-onset 1999–2017 when individually matched to population controls by sex, age, residency and ethnic ancestry. The analyses are stratified by classification criteria applied. ACR-97: 1997 ACR classification criteria for SLE; EA-2019: 2019 EULAR/ACR classification criteria for SLE; SMR: standardized mortality rates

We observed a higher proportion of deaths among males than females (*P*-value <0.001), but median age at diagnosis and subsequently age at death was higher in males (Supplementary Table S1, available at *Rheumatology* online). Among the adult-onset incident cases, SMR was increased by 1.8 (95% CI 1.6–2.2) compared with matched controls (Table 2). SMR did not differ between males and females. The increase in SMR was evident across groups defined by age at diagnosis and ethnic ancestry (Table 2 and Fig. 1). Among cases aged 16–40 years at diagnosis, SMR was similar in European and non-European ancestry subsets [SMR 3.0 (95% CI 1.9–4.7) *vs* SMR 2.7 (95% CI 0.7–8.3)].

**Figure 2.**
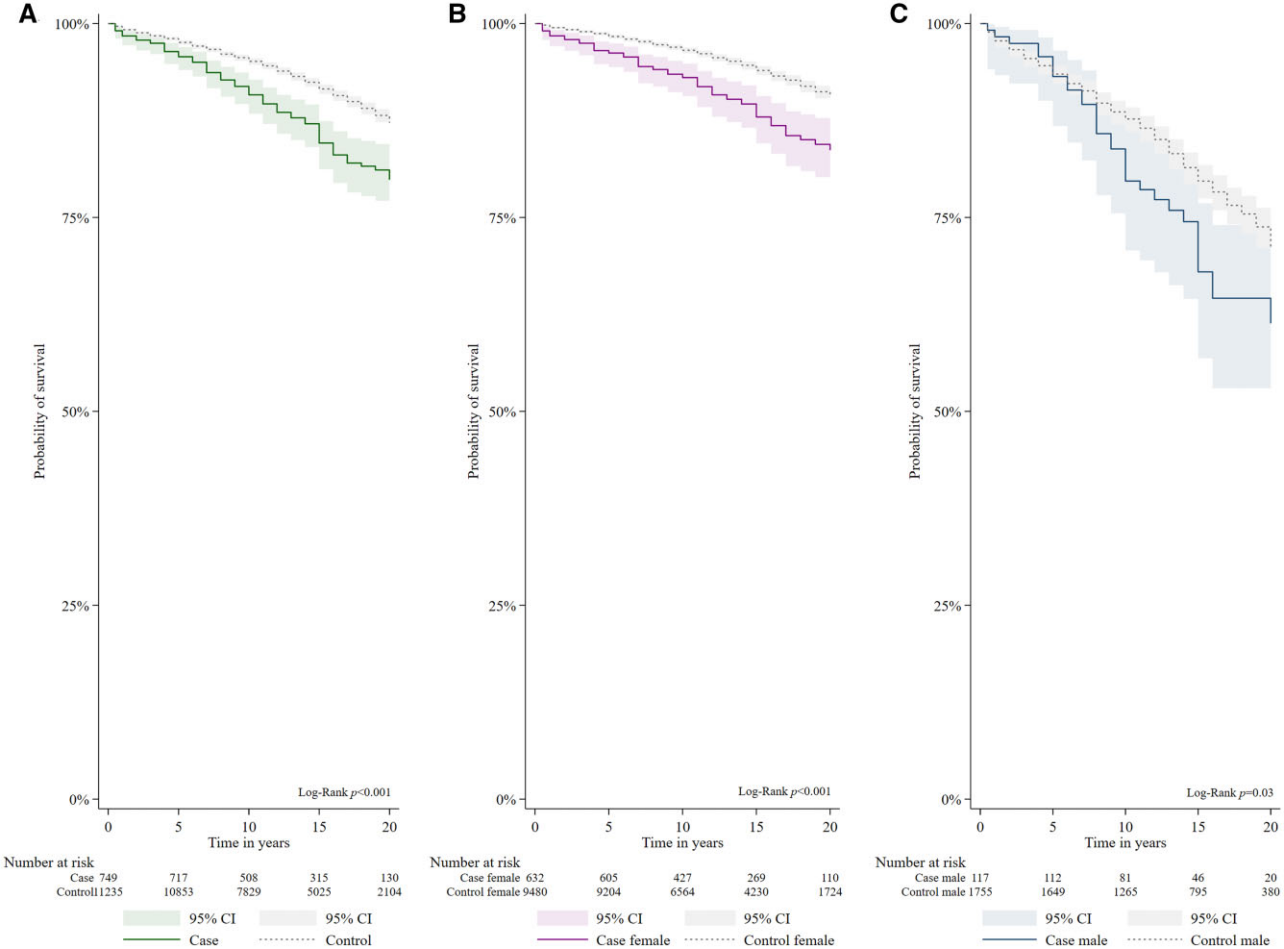
Estimated 20-year survival in new-onset SLE *vs* matched controls in all (**A**), females (**B**) and males (**C**). All cases had disease-onset 1999–2017 and fulfilled the 2019 EULAR/ACR classification criteria for SLE. Controls were individually matched per case by sex, age, residency and ethnic ancestry

The survival rate was lower than in matched controls after 5 years (96.4%% *vs* 98.0%, *P*-value = 0.002), 10 years (92.8% *vs* 95.5%, *P*-value <0.001), 15 years (86.8% *vs* 92.2%, *P*-value <0.001) and 20 years (80.5% *vs* 87.6%, *P*-value <0.001) (Fig. 2). There were no significant difference in 5- and 10-year survival in cases diagnosed 1999–2008 and 2009–17 (*P*-value = 0.473 and *P*-value = 0.8337). Survival differed significantly between male and female cases (*P*-value <0.001) (Fig. 2, Supplementary Table S3, available at *Rheumatology* online).

Older age and LN at diagnosis were associated with reduced mortality both in univariable and multivariable analysis (Table 3). Female sex and use of anti-malaria medication use at diagnosis were protective in univariable analyses, but lost significance in multivariable analysis.

**Table 3.** Association between case descriptive at baseline and mortality in new-onset SLE cases 2000–17

			Univariable analysis		Multivariable analysis		
	Cases, *n*	Deaths, *n*	Unadjusted HR (95% CI)	*P*-value	Adjusted HR^a^ (95% CI)	*P*-value	Harrells c-index
Total	700	93					
Sex							
Male	108	29	1.0		1.0		
Female	592	64	0.4 (0.3–0.6)	<0.001	1.0 (0.6–1.6)	0.983	
Ethnic ancestry							
European	575	87	1.0				
Non-European	125	6	0.3 (0.2–0.8)	0.012	1.1 (0.5–2.6)	0.846	
LN at baseline^b^							
Absent	511	64	1.0				
Present	189	29	1.4 (0.9–2.2)	0.118	2.1 (1.3–3.3)	0.002	
Age at diagnosis (per 5 years)	700	93	1.5 (1.4–1.7)	<0.001	1.6 (1.4–1.7)	<0.001	0.8332
Descriptive at baseline							
Active immunologic disease^c^							
Absent	128	29	1.0		1.0		
Present	568	64	0.6 (0.4–0.9)	0.001	0.8 (0.5–1.2)	0.316	0.8373
Anti-malaria drugs							
Never used	148	36	1.0		1.0		
Before and/or now	475	49	0.4 (0.3–0.6)	<0.001	0.7 (0.5–1.2)	0.205	0.8426
Time period							
Diagn. 2009–17	375	62	1.0				
Diagn. 2000–08	325	31	1.0 (0.6–1.7)	0.848	1.2 (0.7–1.9)	0.556	0.8351

All cases fulfilled the 2019 EULAR/ACR classification criteria for SLE (EA-2019).

a Adjusted for sex, ethnic ancestry, LN and age at diagnosis.

b LN by the EULAR/ACR classification criteria for SLE.

c Anti-dsDNA antibodies or/and anti-Smith antibodies and/or low complement (C3 and/or C4). *n*: number; HR: hazard ratio.

### Mortality in the total SLE cohort

Altogether, the Nor-SLE cohort included 1558 incident and non-incident cases with diagnosis confirmed by chart-review. Of these, 1300 fulfilled the ACR-97 criteria and were defined as the total SLE cohort (Table 1 and Supplementary Fig. S1, available at *Rheumatology* online).

In the total SLE cohort we saw similar sex-specific differences in patient characteristics as those in the incident cohort (Supplementary Table S1, available at *Rheumatology* online). During the observation period, 301/1300 (23%) died, of which 36 (12%) below the age of 50 years. Deaths were more frequent among males than females (*P*-value <0.001), but median age at death was similar (Supplementary Table S1, available at *Rheumatology* online). 

The overall SMR for the total cohort was increased by 2.3 (95% CI 1.5–4.0) compared with matched controls (Table 2). The SMR did not differ between males and females, either in the total cohort or in the subsets defined by age at SLE diagnosis or ethnic ancestry (Supplementary Tables S2 and S4, available at *Rheumatology* online). The age-specific SMR was highest in the juvenile subset (see below) and fell with increasing age (Supplementary Table S2, available at *Rheumatology* online).

### Mortality in the juvenile subset of the total SLE cohort

In the total SLE cohort, there were 93 cases (7%) with juvenile SLE. The SMR in the juvenile SLE subset was higher than in adult-onset subset [SMR 7.2 (95% CI 3.3–14) *vs* SMR 2.2 (95% CI 2.0–2.5); Supplementary Table S4, available at *Rheumatology* online].

The juvenile cases had higher frequency of LN compared with the adult cases [60/93 (65%) *vs* 414/1207 (34%), *P* < 0.001] but did not differ regarding proportion of females [78/93 (84%) *vs* 1049/1207 (87%), *P* = 0.406] or European ancestry cases [77/93 (83%) *vs* 1064/1207 (88%), *P* = 0.129]. When we grouped the juvenile cases by time of diagnosis, we found that age of diagnosis was comparable across time periods, while frequency of LN decreased (Table 4). Twelve of the juvenile SLE cases (12%) died during the observational period, of which six were from 1999–2008. Disease duration at death was longer in juvenile *vs* adult-onset disease (31 *vs* 19 years). Age at death ranged from 23 to 77 years but 75% (9/12) died before the age of 50 years.

**Table 4.** Demographic, clinical features and outcome parameters in juvenile-onset SLE by year of diagnosis

	Juvenile SLE	Matched controls	Juvenile SLE by time of diagnosis					
Year of diagnosis	1950–2017	n/a	1950–69	1970–79	1980–89	1990–99	2000–09	2010–17
Number of cases	93	1395	3	18	14	20	16	22
Baseline demographics								
Female, *n* (%)	78 (84)	1170 (84)	3 (100)	15 (83)	11 (79)	18 (90)	14 (88)	17 (77)
Of European ancestry, *n* (%)	77 (83)	1155 (83)	3 (100)	18 (100)	11 (79)	17 (85)	10 (63)	18 (82)
Age at diagnosis, years µ (s.d.)	13 (1.9)	n/a	14 (1.0)	13 (2.4)	13 (2.2)	13 (2.1)	14 (0.8)	13 (1.7)
Clinical features								
LN^a^, *n* (%)	60 (65)	n/a	2 (67)	15 (83)	10 (71)	13 (65)	10 (63)	10 (46)
Outcome parameters								
Follow-up years^b^, µ (s.d.)	17 (7)	18 (6)	17 (5)	17 (8)	21 (6)	22 (5)	18 (2)	8 (2)
Deaths, *n* (%)	12 (13)	27 (2)	1 (33)	7 (39)	3 (21)	1 (5)	0	0
Disease duration at death, years µ (s.d.)	31 (14)	n/a	n/a	32 (8.3)	28 (12)	n/a	n/a	n/a
Age at death, years median (IQR)	45 (36–56)	53	n/a	43 (36-56)	45 (29–49)	n/a	n/a	n/a

We applied the 1997 ACR classification criteria (ACR-97) for SLE to all juvenile onset SLE cases (diagnosis <16 years of age) living in the study area 1999–2017 and grouped them by year of diagnosis. Each SLE case were individually matched to 15 population controls by sex, age, residential area and ethnic ancestry.

a LN by the 1997 ACR classification criteria for SLE.

b From 1999 or, after 1999; from year of relocation to study area or year of diagnosis. *n*: number; µ: mean; IQR: interquartile range; n/a: not applicable.

We found that males and females with juvenile SLE had SMR of 6.3 (95% CI 0.6–38) and 7.4 (95% CI 3.4–16), respectively. In juvenile SLE, presence of LN increased the SMR to 9.2 (95% CI 3.6–22). Correspondingly, in non-LN the SMR was 4.3 (95% CI 0.8–16).

## Discussion

The mortality gap between SLE patients and controls is a major concern and it is critical to know how the gap evolves across time, sex, ethnicity and age at diagnosis. In this prospective observational SLE cohort study, the relatively high number of cases and the long observation period allowed for robust mortality gap estimates, even in small cohort subsets like juvenile-onset SLE and male SLE. Key and new findings were: (i) the excess mortality in adult-onset SLE persists, (ii) alarmingly high mortality rates in juvenile-onset SLE compared with adult-onset disease and (iii) equally wide mortality gaps in the male and female SLE populations compared with controls.

The recently introduced EA-2019 classification criteria for SLE appear more sensitive than the widely used the ACR-97 criteria [18]. Here, we applied both sets of criteria to new-onset SLE and found that the resulting cohorts were comparable regarding frequency of LN and nearly identical regarding SMR and survival. This observation is important as it indicates possibilities for comparing outcome of SLE across new and old classification criteria, facilitating long-term time trend analyses.

To optimize gap analyses across cohort subsets, we benchmarked the outcome of each individual SLE case against 15 carefully matched controls drawn from the general population. Using this approach, we found that the overall mortality rate of classified new SLE cases resident in Southeast Norway from 1999–2017 was 1.8 times higher than that of the background population. In comparison, the SMR was higher in the total Nor-SLE cohort (2.3), maybe due to its inclusion of non-incident cases with longer disease duration and a greater risk of dying as a result of accumulated damage [19]. Comparable overall mortality rates have recently been reported from population-level studies set in areas with similar case ancestry distribution [20–26]. Some of these studies had less stringent controls, possibly indicating that the high-resolution matching performed in this study is important for analyses of cohort subsets.

Only few studies have estimated 20-year survival of new-onset SLE at population level, but figures range from 53% in Denmark 1975–95 to 84.5% in Hong Kong 1995–2018 [8, 22, 24, 27]. The survival rate in the Hong Kong study is comparable to our study, despite differences in LN frequency [8]. The low survival rate in the Danish study aligns with low 10- and 15-year survival rates in other studies published prior to 1999 [27–31], supporting that the long-term outcome of SLE has improved over time. We report a relatively good 5-year survival in new-onset SLE, indicating effective management of the initial phases of the disease, but the survival gap in SLE compared with controls increases with time from diagnosis. The stable values of overall survival across the study period indicate that the therapies introduced over the last two decades have not yet influenced long-term outcomes. The considerable gap in 20-year survival between SLE and controls highlights the persistent challenge of long-term morbidity prevention.

As expected, we found lower mortality rates than in more ethnically diverse population-based studies from the USA and Asia [32–35]. Even though the SMR among the total population of non-European ancestry cases was high, it is noteworthy that in the subset of cases diagnosed before the age of 50 years, the SMR in the non-European ancestry cases was comparable to that of European cases. Furthermore, non-European ancestry was not associated with death in multivariable analysis. Hence, at least in this study, it appears that age at onset, rather than ethnic background, is a primary factor influencing the mortality gap in SLE.

Our finding of a wider mortality gap in cases with LN than in those without, and increased mortality of LN cases in multivariate analysis underlines that LN is a severe complications of SLE that warrants prompt follow-up [20]. In line with other studies, we found that males with SLE developed LN more frequently than females [36]. Apparently, survival was decreased in male SLE compared with female SLE, but analyses of the controls showed that this only reflected a poorer survival of males in general. This finding illustrates the importance of using of matched controls for survival analyses. The finding of equal mortality gaps in male and female SLE is in line with data from smaller population-based cohorts [21–23, 25, 28, 34, 35].

There were no deaths among the juvenile-onset SLE cases incident in the years 1999–2017, underscoring that early deaths from juvenile-onset SLE occur very rarely, at least in high-income countries [37]. However, in separate analysis of the total SLE cohort, mortality rates were highest in cases diagnosed at young age, similar to previous large tertiary-centre cohorts [38, 39]. Of concern, the SMR of 7.2 in juvenile-onset SLE was twice as high as the SMR reported for juvenile-onset type 1 diabetes in Norway [40]. As expected, we found a higher proportion of LN cases in juvenile-onset SLE [41], but this alone does not fully explain the excess mortality, as juvenile SLE without LN had an SMR >4, albeit with wide CI due to low sample size. Another possible explanation could be the high degree of damage accrual due to many years of disease and medication in juvenile-onset SLE [42, 43]. Additionally, the transition from child to adult may create compliance issues [44]. Previous studies reported mortality rates for juvenile-onset SLE as high as 16–67, but due to differences in study settings and design, these rates may not be directly comparable to ours [12, 28, 45]. Importantly, given that the median time from diagnosis to death in juvenile-onset SLE was 31 years, 20 years of observation is obviously not sufficient to assess mortality in incident juvenile SLE cohorts.

Our study has several strengths. The use of manual SLE case verification by medical experts and matched population controls is labour intensive but adds quality and improves resolution. The study is set in a health system that prioritizes early referral from primary care and offers specialist healthcare for SLE throughout the disease progression, and has mandatory recording of all individual visits by ICD-10. This makes it highly feasible to identify all SLE patients in the study area. The compulsory unique identity number and full coverage of the NCoDR prevents against loss of follow-up. The size of cohort and the long observation time secure robust results, which should be representative of the general population of SLE patients in Norway.

Limitations of our study should be considered. The data from total SLE cohort are skewed by a prevalence-incidence bias, as we select the SLE patients that survive through 1999 and miss patient that died before 1999. Thus, the estimated SMR in this cohort may represent a minimum estimate. We did not have complete data on disease activity or information on organ damage or factors associated with compliance. Therefore, we were not able to study the association between disease activity and mortality and between cumulative organ damage and risk of death. The setting of the cohort in a rather homogeneous European population limits generalizability to other populations.

In conclusion, this contemporary study provides valuable insights into the long-term outcome of SLE at population level. Although the short-term prognosis in SLE is good, the excess mortality in SLE persists and subsequently increases after diagnosis. The width of the mortality gap in juvenile-onset SLE is alarming and highlights the need for optimal management of this high-risk population. Finally, we clearly demonstrate that sex does not influence the width of the mortality gap in SLE.

## Supplementary Material

kead519_Supplementary_Data

## Data Availability

The data underlying this article will be shared on reasonable request to the corresponding author.
