# Supplementary material for: Persisting mortality gap in systemic lupus erythematosus; a population-based study on juvenile- and adult-onset SLE in Norway 1999–2022
Source: Rheumatology (Oxford). 2023 Sep 28;63(8):2109–17. doi: 10.1093/rheumatology/kead519 (PMC11292052; doi:10.1093/rheumatology/kead519)
Supplement: kead519_Supplementary_Data [file kead519_supplementary_data.docx]

Supplementary Material

Reppe Moe, et al. “Persisting mortality gap in Systemic Lupus Erythematosus; a population-based study on juvenile- and adult-onset SLE in Norway 1999-2022”

| Supplementary Figure S1. Flowchart of case inclusion and segregation of subset cohorts and classification criteria applied.  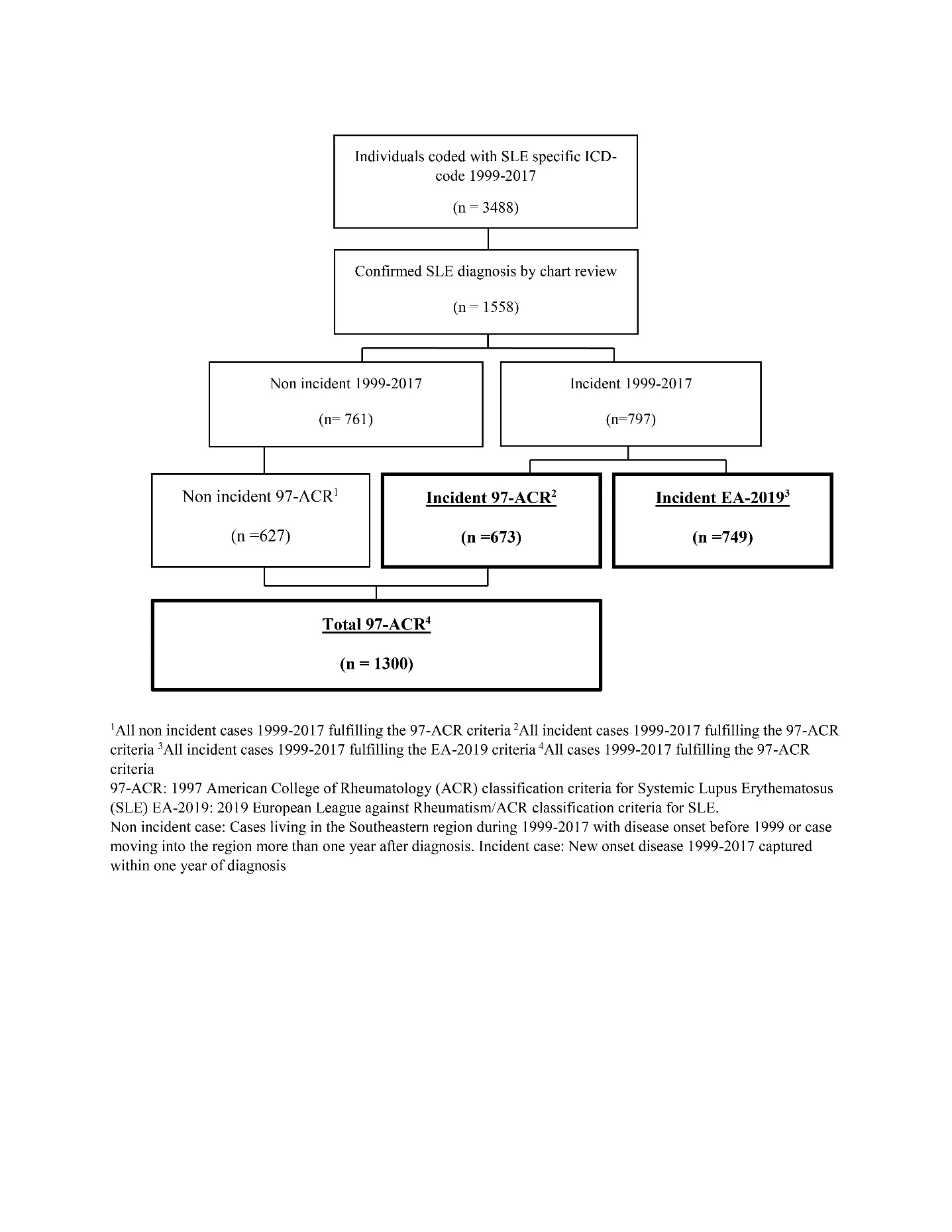  ^1^All non incident cases 1999-2017 fulfilling the 97-ACR criteria ^2^All incident cases 1999-2017 fulfilling the 97-ACR criteria ^3^All incident cases 1999-2017 fulfilling the EA-2019 criteria ^4^All cases 1999-2017 fulfilling the 97-ACR criteria  97-ACR: 1997 American College of Rheumatology (ACR) classification criteria for Systemic Lupus Erythematosus (SLE) EA-2019: 2019 European League against Rheumatism/ACR classification criteria for SLE.  Non incident case: Cases living in the Southeast Norway during 1999-2017 with disease onset before 1999 or case moving into the region more than one year after diagnosis. Incident case: New onset disease 1999-2017 captured within one year of diagnosis  Supplementary Table S1. Baseline demographic, characteristics and outcome in Systemic Lupus Erythematosus, stratified by sex and case cohort | | | | | | | |
| --- | --- | --- | --- | --- | --- | --- | --- |
|  |  | **Total cohort** | | **Incident cohort** | | | |
|  | | **Female** | **Male** | **Female** | **Male** | **Female** | **Male** |
| **Criteria applied** | | ACR-97^a^ | | ACR-97^a^ | | EA-2019^b^ | |
| **Number of cases, n** | | 1123 | 177 | 573 | 100 | 632 | 117 |
| **Baseline demographic** | |  |  |  |  |  |  |
| Age at diagnosis, years µ (SD) | | 35 (15.1) | 41 (18.3) | 37 (15.6) | 44 (18.0) | 38 (15.6) | 46 (17.8) |
| European ancestry, n (%) | | 977 (87) | 164 (93) | 468 (82) | 90 (90) | 517 (82) | 106 (91) |
| Non-European ancestry, n (%) | | 146 (13) | 13 (8) | 105 (18) | 10 (10) | 115 (18) | 11 (9) |
| Juvenile-onset^c^, n (%) | | 78 (7) | 15 (8) | 30 (5) | 7 (7) | 32 (5) | 7 (6) |
| **Clinical characteristics** | |  |  |  |  |  |  |
| Lupus nephritis^d^, n (%) | | 380 (34) | 92 (52) | 171 (30) | 51 (51) | 182 (29) | 58 (50) |
| Cumulative ACR criteria^a^, µ (SD) | | 5.4 (1.3) | 5.3 (1.3) | 5.3 (1.2) | 5.1 (1.2) | 5.0 (1.4) | 4.7 (1.4) |
| **Outcome parameters** | |  |  |  |  |  |  |
| Follow-up years^e^, µ (SD) | | 16 (6.8) | 15 (6.6) | 13 (5.7) | 13 (5.5) | 13 (5.8) | 13 (5.8) |
| Deaths, n (%) | | 237 (21) | 64 (34) | 58 (10) | 25 (25) | 72 (11) | 34 (29) |
| Disease duration at death, years µ (SD) | | 22 (12.8) | 18 (10.8) | 11 (6.4) | 11 (5.4) | 10 (6.4) | 10 (5.3) |
| Age at death, years median (IQR) | | 69 (57-76) | 72 (57-78) | 73 (61-80) | 72 (56-81) | 71 (60-79) | 76 (60- 82) |
|  | |  |  |  |  |  |  |
| n: numbers, µ: mean, SD: standard deviation, IQR: interquartile range, na: not applicable, ^a^The 1997 American College of Rheumatology classification criteria for Systemic Lupus Erythematosus, ^b^2019 European League against Rheumatism/American College of Rheumatology classification criteria for Systemic Lupus Erythematosus,^c^Disease onset before age 16, ^d^Lupus nephritis by the 1997 American College of Rheumatology classification criteria for Systemic Lupus Erythematosus, ^e^From 1999 or, after 1999; from year of relocation to study area or year of diagnosis^.^ | | | | | | | |

Supplementary Table S2. Age-specific standardized mortality rate in Systemic Lupus Erythematosus, stratified by sex and case cohort

| **Total cohort by ACR-97ͣ (n=1300)** | | | | | | |
| --- | --- | --- | --- | --- | --- | --- |
| **Age at diagnosis,** | **All** | | **Female** | | **Male** | |
| **years** | **SMR** | **95% CI** | **SMR** | **95% CI** | **SMR** | **95% CI** |
| <16 | 7.2 | 3.3-14.8 | 7.4 | 3.1-16.4 | 6.3 | 0.6-38.4 |
| 16-29 | 3.8 | 2.8-5.2 | 3.7 | 2.6-5.0 | 6.1 | 2.3-14.1 |
| 30-39 | 2.9 | 2.0-3.9 | 2.8 | 1.9-4.0 | 3.2 | 1.3-6.9 |
| 40-49 | 3.1 | 2.3-4.1 | 3.0 | 2.2-4.1 | 3.6 | 1.6-6.7 |
| 50-59 | 2.0 | 1.5-2.7 | 2.1 | 1.5-3.0 | 1.7 | 0.9-3.0 |
| 60-69 | 1.8 | 1.3-2.5 | 2.2 | 1.5-3.1 | 1.3 | 0.7-2.3 |
| 70-79 | 1.4 | 0.8-2.1 | 1.5 | 0.8-2.5 | 1.2 | 0.4-2.6 |
| ≥80 | 1.0 | 0.4-2.2 | 1.1 | 0.8-2.5 | 0.8 | 0.1-3.0 |
| **Incident cohort by ACR-97ͣ (n=673)** | | | | | | |
| **Age at diagnosis,** | **All** | | **Female** | | **Male** | |
| **years** | **SMR** | **95% CI** | **SMR** | **95% CI** | **SMR** | **95% CI** |
| <16 | na | na | na | na | na | na |
| 16-29 | 1.9 | 0.4-6.1 | 1.9 | 0.2-5.8 | 5.0 | 0.1-62 |
| 30-39 | 2.5 | 0.7-6.4 | 2.5 | 0.5-6.1 | 7.5 | 0.1-144 |
| 40-49 | 2.9 | 1.6-4.9 | 2.9 | 1.1-4.8 | 4.1 | 0.4-10 |
| 50-59 | 1.8 | 1.0-2.9 | 1.8 | 0.9-3.4 | 1.6 | 0.5-4.0 |
| 60-69 | 2.0 | 1.2-3.0 | 2.4 | 1.4-3.9 | 1.2 | 0.4-2.8 |
| 70-79 | 1.3 | 0.7-2.3 | 1.3 | 0.6-2.8 | 1.1 | 0.3-2.9 |
| ≥80 | 1.1 | 0.3-2.7 | 1.1 | 0.3-3.9 | 0.8 | 0.1-3.0 |
| **Incident cohort by EA-2019**ᵇ **(n=749)** | | | | | | |
| **Age at diagnosis,** | **All** | | **Female** | | **Male** | |
| **years** | **SMR** | **95% CI** | **SMR** | **95% CI** | **SMR** | **95% CI** |
| <16 | na | na | na | na | na | na |
| 16-29 | 2.9 | 0.9-7.6 | 1.90 | 0.4-6.4 | 10.70 | 0.9-94 |
| 30-39 | 3.1 | 1.2-7.3 | 3.0 | 1.1-7.3 | 5.00 | 0.1-62 |
| 40-49 | 3.0 | 1.7-5.1 | 2.70 | 1.3-5.1 | 4.10 | 1.4-10.3 |
| 50-59 | 2.0 | 1.2-3.2 | 2.30 | 1.3-3.9 | 1.40 | 0.3-3.8 |
| 60-69 | 1.9 | 1.2-2.8 | 2.50 | 1.5-4.1 | 1.10 | 0.4-2.2 |
| 70-79 | 1.4 | 0.9-2.2 | 1.70 | 1.0-2.8 | 1.30 | 0.6-2.4 |
| ≥80 | 1.3 | 0.5-2.9 | 1.80 | 0.5-4.7 | 0.80 | 0.2-2.6 |
|  |  |  |  |  |  |  |

na: not applicable, SMR: standardized mortality rate, CI: confidence interval ͣthe 1997 American College of Rheumatology classification criteria for Systemic Lupus Erythematosus, ᵇ2019 European League against Rheumatism/American College of Rheumatology classification criteria for Systemic Lupus Erythematosus

Supplementary Table S3. Estimated survival in new-onset Systemic Lupus Erythematous and matched-controls; by classification criteria applied.

|  | **ACR-97ͣ** | | **EA-2019** ᵇ | |
| --- | --- | --- | --- | --- |
|  | **Case** | **Control** | **Case** | **Control** |
| Numbers of cases | 673 | 10 095 | 749 | 11 235 |
| Deaths, n (%) | 83 (12) | 590 (9) | 106 (14) | 914 (8) |
| Follow-up time^c^, µ (SD) | 13.4 (6.7) | 13.5 (5.8) | 13.1 (5.8) | 13.4 (5.8) |
| **Total** |  |  |  |  |
| 5-year survival (95% CI) | 0.97 (0.96-0.98) | 0.98 (0.98 -0.99) | 0.96 (0.95-0.98) | 0.98 (0.98-0.98) |
| 10-year survival (95 % CI) | 0.94 (0.92-0.96) | 0.96 (0.96-0.97) | 0.93 (0.90-0.95) | 0.96 (0.95-0.95) |
| 15-year survival (95 % CI) | 0.87 (0.86-0.90) | 0.93 (0.93-0.94) | 0.87 (0.84-0.89) | 0.92 (0.92-0.93) |
| 20-year survival (95 % CI) | 0.82 (0.78-0.86) | 0.89 (0.88-0.90) | 0.81 (0.76- 0.84) | 0.88 (0.87-0.89) |
| **Female** |  | . |  |  |
| 10-year survival (95 % CI) | 0.95 (0.93 -0.97) | 0.97 (0.97-0.98) | 0.94 (0.91-0.95) | 0.97 (0.97-0.97) |
| 15-year survival (95 % CI) | 0.91 (0.88-0.97) | 0.95 (0.94-0.95) | 0.89 (0.86-0.92) | 0.96 (0.94-0.95) |
| 20-year survival (95 % CI) | 0.85 (0.81-0.90) | 0.91 (0.90-0.92) | 0.84 (0.80-0.87) | 0.91 (0.90-0.92) |
| **Male** |  |  |  |  |
| 10-year survival (95 % CI) | 0.88 (0.80-0.93) | 0.91 (0.89-0.92) | 0.84 (0.75-0.89) | 0.88 (0.86-0.89) |
| 15-year survival (95 % CI) | 0.79 (0.68-0.87) | 0.86 (0.83-0.88) | 0.74 (0.64-0.82) | 0.81 (0.79-0.83) |
| 20-year survival (95 % CI) | 0.67 (0.53-0.77) | 0.78 (0.75-0.81) | 0.64 (0.52-0.74) | 0.73 (0.70-0.76) |

n: numbers, µ: mean, SD: standard deviation CI: confidence interval ͣthe 1997 American College of Rheumatology classification criteria for Systemic Lupus Erythematosus, ᵇ2019 European League against Rheumatism/American College of Rheumatology classification criteria for Systemic Lupus Erythematosus, ^c^From 1999 or, after 1999; from year of relocation to study area or year of diagnosis

Supplementary Table S4. SMR in SLE cases living in the study area 1999-2017 compared to the matched-controls by case characteristics

|  | **SLE** | **Matched**  **Controls** |  |
| --- | --- | --- | --- |
| **Cases/controls** | 1300 | 19000 |  |
|  | **Deaths, n** | **Death, ns** | **SMR**  **(95 % CI)** |
| **Total** | 301 | 2109 | 2.3 (2.0-2.3) |
| **Adult-onset SLE^a^** |  |  |  |
| Total | 289 | 2082 | 2.2 (2.0-2.5) |
| Female | 227 | 1541 | 2.4 (2.0-2.7) |
| Male | 62 | 541 | 1.9 (1.5-2.5) |
| Early-onset^b^ | 164 | 816 | 3.2 (2.7-3.8) |
| Late-onset^c^ | 125 | 1266 | 1.7 (1.4-2.0) |
| **Juvenile-onset SLE^d^** |  |  |  |
| Total | 12 | 27 | 7.2 (3.3-15) |
| **Ancestry** |  |  |  |
| European | 291 | 2069 | 2.3 (2.0-2.6) |
| Non-European | 10 | 40 | 3.7 (1.7-7.5) |
| **Lupus nephritis^e^** |  |  |  |
| Absent | 178 | 1535 | 1.8 (1.6-2.1) |
| Present | 123 | 574 | 3.6 (2.9-4.3) |
|  |  |  |  |

n: numbers; SMR: standardized mortality rate, CI: confidence interval, ^a^Diagnosis ≥16 years of age, ^b^Diagnosis between the age 16-49, ^c^Diagnosis ≥50 years of age, ^d^Diagnosis before age 16, ^e^Lupus nephritis by the 1997 American College of Rheumatology classification criteria for Systemic Lupus Erythematosus

We applied the 1997 American College of Rheumatology classification criteria (ACR-97) for Systemic Lupus erythematosus to all SLE cases living in the study area 1999-2017 (Total cohort). Each SLE case were individually matched to 15 population controls by sex, age, residential area and ethnic ancestry
